# Enhanced functionalization of Mn_2_O_3_@SiO_2 _core-shell nanostructures

**DOI:** 10.1186/1556-276X-6-169

**Published:** 2011-02-24

**Authors:** Sonalika Vaidya, Pallavi Thaplyal, Ashok Kumar Ganguli

**Affiliations:** 1 Department of Chemistry, Indian Institute of Technology, Hauz Khas, New Delhi 110016, India

## Abstract

Core-shell nanostructures of Mn_2_O_3_@SiO_2_, Mn_2_O_3_@amino-functionalized silica, Mn_2_O_3_@vinyl-functionalized silica, and Mn_2_O_3_@allyl-functionalized silica were synthesized using the hydrolysis of the respective organosilane precursor over Mn_2_O_3 _nanoparticles dispersed using colloidal solutions of Tergitol and cyclohexane. The synthetic methodology used is an improvement over the commonly used post-grafting or co-condensation method as it ensures a high density of functional groups over the core-shell nanostructures. The high density of functional groups can be useful in immobilization of biomolecules and drugs and thus can be used in targeted drug delivery. The high density of functional groups can be used for extraction of elements present in trace amounts. These functionalized core-shell nanostructures were characterized using TEM, IR, and zeta potential studies. The zeta potential study shows that the hydrolysis of organosilane to form the shell results in more number of functional groups on it as compared to the shell formed using post-grafting method. The amino-functionalized core-shell nanostructures were used for the immobilization of glucose and L-methionine and were characterized by zeta potential studies.

## Introduction

Surface modification is an integrated and crucial part of material processing and is the basis for the functionality of the material. These functional groups provide further accessibility for anchoring other substrates (or complexes), such as biomolecules or metal ions, into the pores and channels of the carrier material. Surface modification of materials started in early 1990. Badley et al. modified the surface of colloidal silica particles with mercaptopropyl, aminopropyl, and octadecyl chains. Since then modified silica nanoparticles have been utilized for various applications. Silica-coated magnetic nanoparticles modified with γ-mercaptopropyltrimethoxysilane (γ-MPTMS) have been used for solid phase extraction of trace amounts of Cd, Cu, Hg, and Pb [1]. Silanization of silica nanoparticles with 3-MPTMS and with *N*1-[3-(trimethoxysilyl)-propyl]diethylenetriamine has been developed and used for immobilization of oligonucleotides [2] and proteins [3]. Mesoporous vinyl silica was used for the immobilization of penicillin acylase which showed good initial enzymatic activity for the hydrolysis of penicillin G [4,5]. Yoshitake et al. [6] in their studies have shown that the captured transition metal ions on amino-functionalized silica act as adsorption centers for arsenate ions. Surface-functionalized silica particles have found applications in catalysis [7-9], sensors [7,10], and protein immobilization [11,12]. Also, functional groups have been incorporated into silicate surfaces to facilitate molecular imprinting of those surfaces to form highly specific biomimetic catalytic or adsorbent materials [13-15]. Recently, ultrafine silica nanoparticles, with surfaces functionalized by cationic-amino groups, have been shown to not only bind and protect plasmid DNA from enzymatic digestion but also transfect cultured cells and express encoded proteins [16,17].

Two commonly applied methods for the introduction of functional groups onto the silica surface are co-condensation and post-grafting of functional silanes. Both the methods have certain drawbacks associated with them. The post-synthesis grafting method results in inhomogeneity of the functional group on the surface of the nanoparticles. This is because the organic moieties (functional groups) are concentrated near the entries of the mesopores and the exterior surfaces [18]. The second most commonly used for functionalization of nanoparticles is the co-hydrolysis of organosilanes with a tetraalkoxysilicate. Using the co-hydrolysis techniques, silica particles with surface vinyl [19,20], carboxylate [21], amine [22], dihydroimidazole [23], pyridine [15], and quaternary amine [15] have been developed. Co-condensation reactions of organotrialkoxysilanes and TEOS at various molar ratios were carried out by Mann and co-workers [24] to covalently link organo-functionalities such as phenyl, allyl, mercapto, amino, cyano, perfluoro, or dinitrophenylamino moieties to the core-shell nanostructures of Au coated with functionalized silica. However, the main disadvantage of this method is that most of the functional groups may be embedded in the silica network [25].

The above applications of modified silica particles motivated us to synthesize core-shell nanostructures of Mn_2_O_3 _nanoparticles (core) with functionalized silica shell. Silica-coated Mn_2_O_3 _(not functionalized) nanostructures were also synthesized. Mn_2_O_3 _is an antiferromagnetic oxide with the transition temperature of 90 K. It is used as a catalyst in the oxidation of ethylene [26] and methane [27] and in the decomposition of NO_*x *_[28]. Nanocomposites of Mn_2_O_3 _and Mn_3_O_4 _on mesoporous silica showed significant catalytic activity toward CO oxidation below 523 K [29]. The oxidative dehydrogenation of ethane in wet natural gas over Mn_2_O_3_/SiO_2 _catalyst was investigated by Ping et al. [30]. In most of the earlier reports the functionalizing agent is assembled after the formation of the silica shell, or a co-condensation method has been used. In our studies we have optimized the conditions such that the functionalized shell can be formed from the hydrolysis of the respective precursors, i.e., the organotrialkoxysilanes to form amino-, allyl-, and vinyl-functionalized silica shell. To the best of our knowledge there has been only one report on the formation of amino-functionalized silica shell over ultrasmall superparamagnetic iron oxide particles (USPIO) using the hydrolysis of the organosilane. These particles were coated with silica, (3-aminopropyl)trimethoxysilane (3-APTMS), and [*N*-(2-aminoethyl)-3-aminopropyl]trimethoxysilane (AEAPTMS), and their ability to label immortalize progenitor cells for magnetic resonance imaging (MRI) was compared. It was observed that the three coated USPIO particles were biocompatible and were intensely internalized in immortalized progenitor cells which make them a suitable candidate for MR cell-labeling and cell-tracking experiments [31]. Thus, we believe that our methodology will *ensure more functional groups *over the core-shell nanostructures and hence can be used for biological applications in a more efficient way. In this study we also show the ability of these nanostructures to immobilize glucose and L-methionine.

Our methodology, using surfactant, can be used to synthesize silica shell over nanoparticles which are synthesized at high temperature and are not present in colloidal form (have high degree of agglomeration). Our study can also be extended to form silica shell over individual nanoparticles (having high degree of agglomeration) which can then be used in various biomedical and catalytic applications. We have also increased the concentration of functional groups on the surface of core-shell nanostructures with the use of organosilane precursors to form the shells. The methodology is an improvement over the commonly used post-grafting or co-condensation method. This point has been proved in this article by carrying out two studies: one with zeta potential and other using a fluorescamine dye. Thus, the methodology described can be used to synthesize core-shell nanostructures with *high density of functional groups *which can be further used for various analytical purposes such as extraction of trace elements with high specificity. The high density of functional groups will also ensure an increase in the number biomolecules or drugs that can be immobilized on these nanostructures. For this we have carried out a case study using glucose and L-methionine and have shown that the functionalized core-shell nanostructures can be used to immobilize biomolecules.

## Materials and methods

Mn_2_O_3 _nanoparticles were synthesized by thermal decomposition of manganese oxalate nanorods [32]. For the synthesis of core-shell nanostructures with silica shell, Mn_2_O_3 _nanoparticles were dispersed in Tergitol/cyclohexane mixture. Silica coating was carried out using hydrolysis of TEOS with ammonia. Amino-functionalized core-shell nanostructures Mn_2_O_3 _nanoparticles were dispersed in Tergitol/1-octanol/cyclohexane mixture followed by hydrolysis of 3-APTMS using ammonia and water. Vinyl- and allyl-functionalized core-shell nanostructures were synthesized by dispersing Mn_2_O_3 _nanoparticles in Tergitol/water system. The functionalized silica shell was grown over the Mn_2_O_3 _nanoparticles by hydrolysis of vinyltrimethoxysilane and allyltrimethoxysilane using ammonia. In order to confirm that the above methodology ensures more functional groups on the core-shell, Mn_2_O_3_@amino-functionalized silica core-shell nanostructures were also synthesized by the post-grafting method wherein Mn_2_O_3 _nanoparticles were dispersed in Tergitol/1-octanol/cyclohexane mixture. Mn_2_O_3 _nanoparticles were coated with silica using TEOS as the shell forming agent followed by addition of 3-APTMS. Amount of amino groups on the core-shell nanostructures with amino-functionalized silica (with and without TEOS) was calculated using fluorescamine dye.

Glucose and L-methionine immobilization was carried out by taking amino-functionalized core-shell nanostructures in phosphate buffer (pH 8) to form a dispersion under sonication. To this, glucose solution was added followed by stirring for 48 h for the immobilization of glucose while L-methionine immobilization was carried out by addition of L-methionine solution followed by stirring for 24 h after which the resultant mixture was heated at 60°C. The above core-shell nanostructures were characterized using powder X-ray diffraction (PXRD), FTIR, HRTEM, surface charge measurement (zeta potential), and fluorescence studies. All the details regarding synthesis and characterization are given in the supporting information.

## Results and discussion

**Figure 1:**
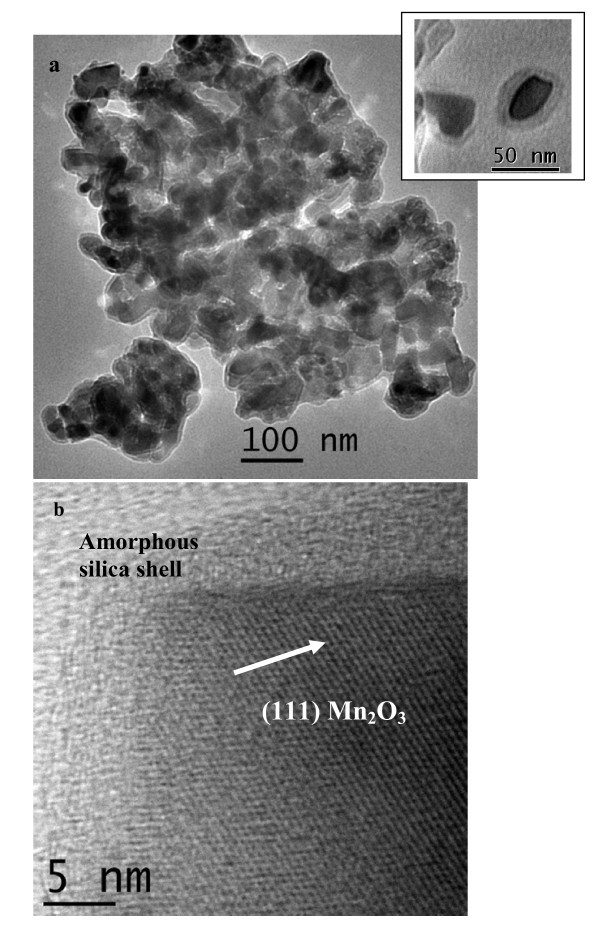
**TEM and HRTEM image**. **(a) **TEM and **(b) **HRTEM images of Mn_2_O_3_@SiO_2 _core-shell nanostructures.

TEM image of Mn_2_O_3_@SiO_2 _core-shell nanostructures shows cores with size ranging from 25 to 100 nm with a shell thickness of 5 nm (Figure 1a). The presence of amorphous silica shell was clearly observed in the TEM image. The synthetic methodology utilizes already synthesized Mn_2_O_3 _nanoparticles which has been prepared from the route known in the literature [32]. HRTEM image (Figure 1b) shows lattice fringes corresponding to (111) plane of Mn_2_O_3_. The amorphous silica shell was clearly observed surrounding the crystalline core in the high resolution TEM image (Figure 1b). Thus HRTEM of Mn_2_O_3_@SiO_2 _core-shell nanostructures confirms the chemical composition of core as Mn_2_O_3 _and shell as amorphous silica.

Mn_2_O_3_@SiO_2 _core-shell nanostructures are present in an aggregated form as observed from TEM images in Figure 1. The presence of aggregates could be attributed to the formation of H-bond between the silica shells due to the presence of Si-OH bond over the shell surface. These Si-OH bonds were formed by the hydrolysis of TEOS in the presence of ammonia and water at room temperature. We have also discussed the aggregation effect in silica-coated core-shell nanostructures in our earlier report [33]. It is also to be noted that the starting material (Mn_2_O_3 _nanoparticles) used for the synthesis of silica shell is a magnetic material, present in powder form. Thus, there is an inherent tendency of these oxide nanoparticles to agglomerate. However, a challenge still remains to form silica shell over individual nanoparticles (for the oxides present in powder form with high degree of agglomeration). The main emphasis in this article is on the enhancement of functional groups on the surface of core-shell nanostructures by using an organosilane precursor to form the shell and compared with our studies of shell formation by the post-grafting method which has been the common procedure in earlier studies [25]. This point has been discussed in later sections.

**Figure 2:**
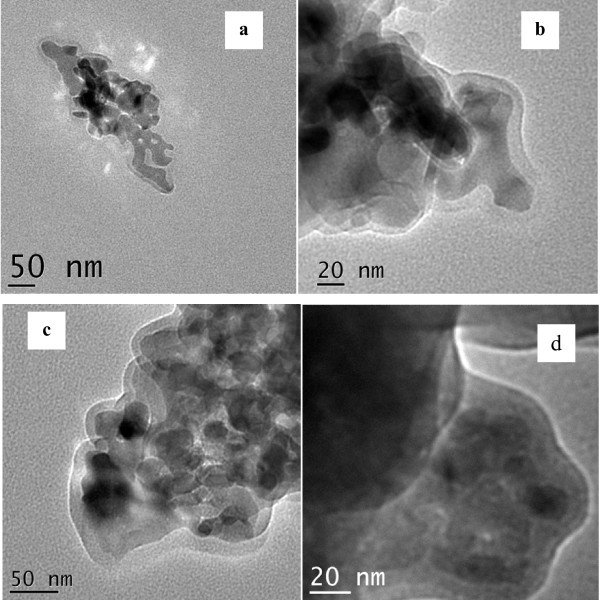
**TEM images of functionalized core-shell**. TEM images of **(a) **Mn_2_O_3_@amino-functionalized silica (without TEOS), **(b) **Mn_2_O_3_@vinyl-functionalized silica, **(c) **Mn_2_O_3_@allyl-functionalized silica, and **(d) **Mn_2_O_3_@amino-functionalized silica (with TEOS).

Figure 2a shows TEM image for Mn_2_O_3_@amino-functionalized silica particles with core diameter of 25-30 nm and shell thickness of 5 nm. Nanoparticles of Mn_2_O_3_@vinyl-functionalized silica (Figure 2b) show core-shell nanostructures with a core diameter of 25-30 nm and shell thickness of 5-10 nm. Cores with diameter of 25-30 nm with a shell thickness of 10-15 nm were observed (Figure 2c) for Mn_2_O_3_@allyl-functionalized silica. It is to be noted that the shell in the above three core-shell nanostructures is formed by the hydrolysis of organosilane precursors, which ensures that these core-shell nanostructures bear the respective functional groups (amine, vinyl, and allyl) on their surface. Core-shell nanostructures (amine groups over the shell) were obtained (Figure 2d) when the synthesis was carried out with TEOS and APTMS. The core size varied from 20 to 25 nm and a shell thickness was found to be 10 nm.

Bands at 3429, 1632, 572, and 520 cm^-1 ^corresponding to O-H stretching, O-H bending, and Mn-O stretching were observed in IR spectrum of Mn_2_O_3 _nanoparticles. Additional bands at 1123 and 1079 cm^-1 ^corresponding to Si-O-Si stretching were observed for the silica-coated nanostructures. This gives further evidence for the coating of silica over Mn_2_O_3 _nanoparticles corroborating with the TEM studies. Table S1 in Additional file 1 summarizes the IR bands for the functionalized core-shell nanostructures. Note that in all the three core-shell nanostructures, Si-O-Si stretching band was observed even though TEOS *was not added*. This confirms that the stretching band was observed due to the functionalized silica shell formed as a result of hydrolysis of the organosilane precursors. Thus, IR spectrum gives us an additional proof for the formation of core-shell nanostructures with functionalized shells. In addition to the above we also observed C=C stretching vibrations in the IR spectrum of vinyl- and allyl-functionalized core-shell nanostructures which also suggest the proper functionalization of the shell.

**Figure 3:**
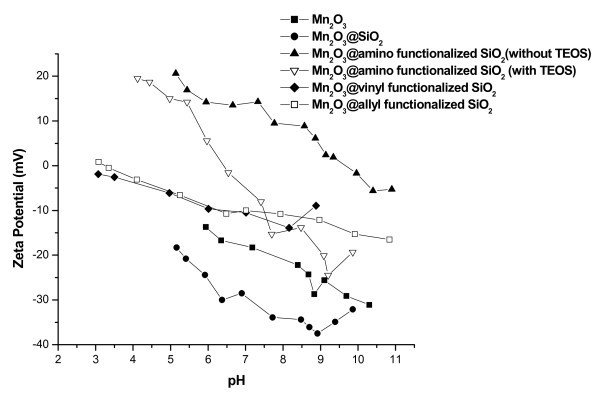
**Zeta potential vs. pH plot**. Zeta potential versus pH plot for bare Mn_2_O_3_, Mn_2_O_3_@SiO_2_, Mn_2_O_3_@amino-functionalized silica (with TEOS), Mn_2_O_3_@amino-functionalized silica (without TEOS), Mn_2_O_3_@vinyl-functionalized silica, and Mn_2_O_3_@allyl-functionalized silica core-shell nanostructures.

Zeta potential studies for uncoated and coated Mn_2_O_3 _nanoparticles were carried out with varying pH (Figure 3). Increase in the negative zeta potential values were observed for the coated particles compared to the uncoated particles, which suggests a uniform coating of silica over Mn_2_O_3 _nanoparticles. The negative surface charge of silica is expected due to the presence of hydroxyl groups on the surface of silica.

Figure 3 shows zeta potential versus pH curves for bare Mn_2_O_3_, Mn_2_O_3_@SiO_2_, Mn_2_O_3_@amino-functionalized silica (with TEOS), Mn_2_O_3_@amino-functionalized silica (without TEOS), Mn_2_O_3_@vinyl-functionalized silica, and Mn_2_O_3_@allyl-functionalized silica core-shell nanostructures. The silica-coated Mn_2_O_3 _bears a negative surface charge at pH > 3. It has been reported in an earlier study [34] that the presence of amine shifts the iso-electric point (IEP) toward higher pH values as the *pKa *of aminopropyl group is 9.8. The amine group is protonated at pH < 9. In Mn_2_O_3_@amino-functionalized silica (without TEOS), the IEP was found to be 9.6 which suggests that the amino groups are present on the surface of the core-shell particles. At pH > IEP, deprotonation of the positively charged R-NH_3_^+ ^groups results in a negative surface charge while the presence of R-NH_3_^+ ^groups at pH < IEP results in a positive surface charge. The zeta potential depends on two main factors viz. pH and concentration of the sample [35]. In our study we have fixed the concentration of the sample from 1 to 2 mg in 10 ml of 10 mM NaCl and have studied the zeta potential as a function of pH.

Zeta potential values are sensitive to the surface charge of the outer particle surface and hence our result suggests that the amine groups are located on the outer surface of the core-shell nanostructures. It is also to be noted that the values of the obtained zeta potential do not refer to a single particle but represent an ensemble of particles present in the system. In order to ensure that more functional groups are present over the shell, zeta potential studies were carried out on Mn_2_O_3_@amino-functionalized silica (with TEOS) wherein amino functionalization was carried out by post-grafting method using APTMS. It was observed that the zeta values were less positive than Mn_2_O_3_@amino-functionalized silica (without TEOS). Zeta values as earlier mentioned are dependent on the surface charge of the outer particle, which suggests that the number of amine groups over the functionalized core-shell nanostructures synthesized using post-grafting method is less than the one synthesized using APTMS as the shell forming agent. The IEP for Mn_2_O_3_@amino-functionalized silica (with TEOS) also shifts to low pH (=6.3), which also suggests the presence of less number of amine groups and more number of hydroxyl groups over the surface of these core-shell nanostructures. The above inference was further confirmed by using fluorescamine dye. The concentration of amine groups was found to be 0.302 μmol/g in the case of Mn_2_O_3_@amino-functionalized silica (without TEOS) and 0.274 μmol/g for Mn_2_O_3_@amino-functionalized silica (with TEOS).

Surface charge density was calculated using Guoy-Chapman equation [36]. The surface charge density was calculated at two pH value viz. 5.4 and 6.5 and was found to be 3.96 mC/m^2 ^(at pH 5.4) and 3.14 mC/m^2 ^(at pH 6.5) for Mn_2_O_3_@amino-functionalized silica (without TEOS). The surface charge density for Mn_2_O_3_@amino-functionalized silica (with TEOS) was found to be 3.31 mC/m^2 ^(at pH 5.4) and -0.37 mC/m^2^. Thus, both calculations (using fluorescamine and zeta potential) suggest that the core-shell nanostructures (amino-functionalized) synthesized using the hydrolysis of 3-APTMS only bear high density of amino groups on the shell as compared to the core-shell nanostructures synthesized using post-grafting method.

The zeta potential of allyl- and vinyl-functionalized silica was higher than that of silica-coated and bare nanoparticles, which also suggests the presence of allyl and vinyl groups on the surface of the core-shell nanostructures.

Zeta potential studies for the amino-functionalized core-shell nanostructures immobilized with glucose and L-methionine were carried out by dispersing the particles in 10 mM NaCl solution (Table 1). The zeta potential values changed from positive to negative suggesting that glucose and L-methionine have been immobilized onto the surface of the core-shell nanostructures. Thus, the change in zeta potential values can be used to detect the immobilization of bio-molecules over nanoparticles. The immobilization of biomolecules (glucose and L-methionine) is just to show the use of functionalized silica core-shell structures for possible applications.

**Table 1:** Zeta potential values for amino-functionalized and bio-molecule immobilized core-shell nanostructures

Sample	Zeta potential (mV)
Mn_2_O_3_@amino-functionalized SiO_2_	29.5
Glucose immobilized Mn_2_O_3_@amino-functionalized SiO_2_	-7.2
L-methionine immobilized Mn_2_O_3_@amino-functionalized SiO_2_	-12.0

## Conclusions

Synthesis of core-shell nanostructures with functionalized silica shell was carried out using the hydrolysis of the organosilane precursors. TEM shows the formation of core-shell with a core diameter of 25-30 nm and a shell nanostructures thickness of 5-15 nm. An increase in (negative) the zeta potential value compared to the bare Mn_2_O_3 _and silica-coated Mn_2_O_3 _core-shell nanostructures also confirms the presence of functional groups over the surface of the core-shell. We have also shown that the hydrolysis of the organosilane precursor results in increased value of the zeta potential and the surface charge density, which confirms more number of functional group over the nanostructures.

## Abbreviations

AEAPTMS: [*N*-(2-aminoethyl)-3-aminopropyl]trimethoxysilane; 3-APTMS (3-aminopropyl)trimethoxysilane; IEP: iso-electric point; γ-MPTMS: γ-mercaptopropyltrimethoxysilane; MRI: magnetic resonance imaging; USPIO: ultrasmall superparamagnetic iron oxide particles.

## Competing interests

The authors declare that they have no competing interests.

## Authors' contributions

SV carried out the synthesis and characterization of core-shell nanostructures. PT assisted in the synthesis of core-shell nanostructures. Basic idea and the execution of the project was carried out under the guidance of AKG. All authors read and approved the final manuscript.

## Supplementary Material

Additional file 1 **Supplemental Material**. A description of the experimental methods, supplementary figures and tables. **Figure S1. The PXRD pattern of Mn_2_O_3_@SiO_2 _core-shell nanostructures**. Reflections corresponding to Mn_2_O_3 _(cubic) with a broad feature in the 2 theta range from 20° to 30° are observed indicating the presence of amorphous silica coated on Mn_2_O_3 _particles. **Figure S2. EDAX spectrum of Mn_2_O_3_@SiO_2 _core-shell nanostructures**. Figure shows peaks corresponding to Mn, O, and Si confirming their presence in the core-shell nanostructures. **Table S1 Details of the IR frequencies for functionalized core-shell nanostructures** Click here for file
