# Supplementary material for: Enhanced functionalization of Mn2O3@SiO2 core-shell nanostructures
Source: Nanoscale Res Lett. 2011 Feb 24;6(1):169. doi: 10.1186/1556-276X-6-169 (PMC3211221; doi:10.1186/1556-276X-6-169)
Supplement: Additional file 1 — Supplemental Material. A description of the experimental methods, supplementary figures and tables. Figure S1. The PXRD pattern of Mn2O3@SiO2 core-shell nanostructures. Reflections corresponding to Mn2O3 (cubic) with a broad feature in the 2 theta range from 20° to 30° are observed indicating the presence of amorphous silica coated on Mn2O3 particles. Figure S2. EDAX spectrum of Mn2O3@SiO2 core-shell nanostructures. Figure shows peaks corresponding to Mn, O, and Si confirming their presence in the core-shell nanostructures. Table S1 Details of the IR frequencies for functionalized core-shell nanostructures [file 1556-276X-6-169-S1.DOC]

**Additional File 1**

**Experimental methods**

For the synthesis of core-shell nanostructures with silica shell, 21 ml of Tergitol was added to 180 ml of cyclohexane. To this, 9 ml of the dispersion of Mn_2_O_3_ nanoparticles in cyclohexane (concentration of nanoparticles: 1 mg/ml) was added with stirring followed by addition of 3.5 ml of liq. NH_3_ (25 wt%) and 30 μl of TEOS. The resultant mixture was allowed to stir for 15 h after which it was centrifuged, washed with acetone and dried.

For the synthesis of amino-functionalized core-shell nanostructures, the following procedure was followed: 21 ml of the neutral surfactant, Tergitol and 15.6 ml of 1-octanol (co-surfactant) was added to 180 ml of cyclohexane. To this, 9 ml of the dispersion of Mn_2_O_3_ nanoparticles (synthesized by thermal decomposition of manganese oxalate nanorods [32]) in cyclohexane (concentration of nanoparticles: 1 mg/ml) was added with stirring followed by addition of 3.5 ml of liq. NH_3_ (25 wt%) and 3.5 ml of water. To this 30 μl of (3-aminopropyl)trimethoxysilane (3-APTMS) was added. The resultant mixture was allowed to stir for 15 h after which it was heated at 60°C for 3 h. The resultant mixture was allowed to cool after which it was stirred for another 15 h. The particles were separated by centrifugation, washed with acetone and dried.

For the synthesis of vinyl- and allyl-functionalized core-shell nanostructures, 2 ml of Tergitol was added to 50 ml water. To this 7 ml of the dispersion of Mn_2_O_3_ nanoparticles in water (concentration of nanoparticles: 1 mg/ml) was added with stirring followed by addition of 3 ml of NH_3_ (25 wt%) for vinyl-functionalized and 25 ml for allyl-functionalized core-shell nanostructures. To this, 100 μl of vinyltrimethoxysilane and allyltrimethoxysilane were added to form vinyl-functionalized and allyl-functionalized core-shell nanostructures, respectively. The resultant mixture was stirred for 15 h after which it was heated at 60°C for 3 h. The resultant mixture was allowed to cool after which it was stirred for another 15 h. The particles were separated by centrifugation, washed with acetone and dried. For synthesis of Mn_2_O_3_@amino-functionalized silica core-shell nanostructures using post-grafting method, 21 ml of the neutral surfactant, Tergitol and 15.6 ml of 1-octanol (co-surfactant) were added to 180 ml of cyclohexane. To this, 9 ml of the dispersion of Mn_2_O_3_ nanoparticles in cyclohexane (concentration of nanoparticles: 1 mg/ml) was added with stirring followed by addition of 3.5 ml of liq. NH_3_ (25 wt%) and 3.5 ml of water. To this 30 μl of TEOS was added and the system was stirred for 15 h after which 30 μl of 3-APTMS was added. The resultant mixture was allowed to stir for 15 h after which it was heated at 60°C for 3 h. The resultant mixture was allowed to cool after which it was stirred for another 15 h. The particles were separated by centrifugation, washed with acetone and dried.

For the determination of the amount of amino groups over core-shell nanostructures, 5 mg of core-shell nanostructure was dispersed in a mixture of 2 ml of NaHCO_3_ solution (5 wt%) and 2 ml ethanol. 2 ml of this dispersion was diluted with 1 ml of NaHCO_3_ solution and 1 ml of water. To this 300 μl of 5 mM fluorescamine solution in methanol was added. The intensity of fluorescence (excited at 420 nm) was measured at 480 nm. For quantifying the number of amino groups on core-shell nanostructures, the procedure was repeated by using 10 μM solution of alanine.

Glucose immobilization was carried out by taking 5 mg of the amino-functionalized core-shell nanostructures in 5 ml of phosphate buffer (pH 8) to form a dispersion under sonication. This dispersion was diluted by adding 15 ml of phosphate buffer. To this 5 ml of 6 mM glucose solution was added. The resultant mixture was allowed to stir for 48 h. The particles were separated using centrifugation and washed with water to remove unattached glucose and phosphate buffer. The particles were dried at 60°C for 10 h.

l-methionine immobilization was carried out by taking 5 mg of the amino-functionalized core-shell nanostructures in 5 ml of phosphate buffer (pH 8) to form a dispersion under sonication. This dispersion was diluted by adding 15 ml of phosphate buffer. To this 5 ml of 10 mM l-methionine solution was added. The resultant mixture was allowed to stir for 24 h followed by heating at 60°C for 3 h. The system was allowed to cool and the particles were separated using centrifugation and washed with water to remove unattached l-methionine and phosphate buffer. The particles were dried at 60°C for 10 h.

**Characterization**

Powder X-ray diffraction (PXRD) studies were carried out on a Bruker D8 Advance diffractometer with Ni-filtered Cu *Kα* radiation. Data were collected with a step size of 0.02° and a scan speed of 1 s per step. Raw data were subjected to background correction and the *Kα*_2_-lines were stripped off. FT-IR spectra were recorded on a Nicolet Protege 460 Fourier transform infrared (FTIR) spectrometer. The powder was made into a disk with KBr and scanned in the range from 400 to 4000 cm^-1^. Transmission electron microscopy (TEM) and energy dispersive X-ray analysis (EDAX) studies were carried out on an FEI Technai G^2^ 20 electron microscope operated at 200 kV. TEM specimens were prepared by dispersing the sample in ethanol by ultrasonic treatment and pouring one drop of the dispersion on a porous carbon film supported on a copper grid which was then dried in air. Zeta potential studies were performed with Zetasizer Nano ZS90 (Malvern Instruments, U.K.). The samples (1 to 2 mg) were dispersed in 10 ml of 10 mM NaCl solution with pH 7. The dispersion was then transferred to a folded capillary cell with gold electrodes. Measurement of zeta potential with variation in pH was carried out on an MPT2 Autotitrator attached to Zetasizer Nano ZS90. The sample was dispersed in 10 mM NaCl solution and the pH of the solution was adjusted by addition of either 10 mM HCl or NaOH solution. Fluorescence studies were carried out on FL 3-11, Fluorolog-3 modular spectrofluorometer (Horiba-Jobin Yvon, Inc, Edison, NJ, USA). The spectrofluorometer contains single Czerny-Turner grating excitation and emission monochromators as wavelength selection devices, 450 W Xe-arc lamp as the excitation source, and PMT as the detector. All data were acquired using 1 cm^2^ path length quartz cuvettes.

**Figure S1. The PXRD pattern of Mn_2_O_3_@SiO_2_ core-shell nanostructures**.

**2-Theta Scale**

**Lin Counts**

70

**222**

**400**

**440**

**622**

Reflections corresponding to Mn_2_O_3_ (cubic) with a broad feature in the 2 theta range from 20° to 30° are observed indicating the presence of amorphous silica coated on Mn_2_O_3_ particles.

**Figure S2. EDAX spectrum of Mn_2_O_3_@SiO_2_ core-shell nanostructures**.

Figure shows peaks corresponding to Mn, O, and Si confirming their presence in the core-shell nanostructures.

**Table S1 Details of the IR frequencies for functionalized core-shell nanostructures**

| Bands | Mn_2_O_3_ (cm^-1^) | Mn_2_O_3_@SiO_2_ (cm^-1^) | Mn_2_O_3_@amino-functionalized SiO_2_ (without TEOS) (cm^-1^) | Mn_2_O_3_@vinyl-functionalized SiO_2_ (cm^-1^) | Mn_2_O_3_@allyl-functionalized SiO_2_ (cm^-1^) |
| --- | --- | --- | --- | --- | --- |
| ν_Mn-O_ stretching | 572, 520 | 572, 520 | 572, 519 | 576, 524 | 577, 528 |
| ν_O-H_ stretching | 3429 | 3429 | 3404 | 3441 | 3432 |
| ν_O-H_ bending/ν_C=C_ stretching | 1632 | 1632 | 1631 | 1639 | 1631 |
| ν_C-H_ stretching | - | - | 2928 | 2924 | 2922 |
| ν_Si-O-Si_ stretching | - | 1123, 1079 | 1123, 1038 | 1115 | 1115 |
